# Multisensor System for Isotemporal Measurements to Assess Indoor Climatic Conditions in Poultry Farms

**DOI:** 10.3390/s120505752

**Published:** 2012-05-04

**Authors:** Eliseo Bustamante, Enrique Guijarro, Fernando-Juan García-Diego, Sebastián Balasch, Antonio Hospitaler, Antonio G. Torres

**Affiliations:** 1 Institute of Animal Science and Technology, Universitat Politècnica de València, Camino de Vera s/n 46022 Valencia, Spain; E-Mail: elbusgar@doctor.upv.es; 2 Department of Construction Engineering and Civil Engineering Projects, Universitat Politècnica de València, Camino de Vera s/n 46022 Valencia, Spain; E-Mail: ahospitaler@cst.upv.es; 3 Department of Electronic Engineering, Universitat Politècnica de València, Camino de Vera s/n 46022, Valencia, Spain; E-Mail: eguijarro@eln.upv.es; 4 Department of Applied Physics, Universitat Politècnica de València, Camino de Vera s/n 46022 Valencia, Spain; E-Mail: fjgarcid@upvnet.upv.es; 5 Center of Physical Technologies, Asociated Unity ICMM-CSIC/UPV, Universitat Politècnica de València, Av. de los Naranjos s/n. 46022 Valencia, Spain; 6 Department of Statistical, Operation Research and Quality, Universitat Politècnica de València, Camino de Vera s/n 46022 Valencia, Spain; E-Mail: sbalasch@eio.upv.es

**Keywords:** poultry building, sensors, air velocity, isotemporal measurements, multipoint measurements, troubleshooting

## Abstract

The rearing of poultry for meat production (broilers) is an agricultural food industry with high relevance to the economy and development of some countries. Periodic episodes of extreme climatic conditions during the summer season can cause high mortality among birds, resulting in economic losses. In this context, ventilation systems within poultry houses play a critical role to ensure appropriate indoor climatic conditions. The objective of this study was to develop a multisensor system to evaluate the design of the ventilation system in broiler houses. A measurement system equipped with three types of sensors: air velocity, temperature and differential pressure was designed and built. The system consisted in a laptop, a data acquisition card, a multiplexor module and a set of 24 air temperature, 24 air velocity and two differential pressure sensors. The system was able to acquire up to a maximum of 128 signals simultaneously at 5 second intervals. The multisensor system was calibrated under laboratory conditions and it was then tested in field tests. Field tests were conducted in a commercial broiler farm under four different pressure and ventilation scenarios in two sections within the building. The calibration curves obtained under laboratory conditions showed similar regression coefficients among temperature, air velocity and pressure sensors and a high goodness fit (R^2^ = 0.99) with the reference. Under field test conditions, the multisensor system showed a high number of input signals from different locations with minimum internal delay in acquiring signals. The variation among air velocity sensors was not significant. The developed multisensor system was able to integrate calibrated sensors of temperature, air velocity and differential pressure and operated succesfully under different conditions in a mechanically-ventilated broiler farm. This system can be used to obtain quasi-instantaneous fields of the air velocity and temperature, as well as differential pressure maps to assess the design and functioning of ventilation system and as a verification and validation (V&V) system of Computational Fluid Dynamics (CFD) simulations in poultry farms.

## 1. Introduction

Broiler production is an essential food industry in many countries. Currently, modern poultry production chains supply safe, nutritious and relatively cheap high-quality protein; for this reason, large-scale commercial poultry production plays nowadays an essential role supplying food to a rapidly growing, urban middle class worldwide [1]. From 2000 to 2010, the production of the poultry meat sector has increased more than 4% per year, compared to 2.1% for pig and 1.1% for beef. As a result, its share in global meat production has increased from 15% in the 1970 decade to 33.5% at present 2010 [2]. This growth has been accompanied and supported by rapid technological, scientific and industrial changes associated with the development of highly industrialized landless intensive systems. Recent developments of poultry meat production have consisted in huge genetic improvements, the use of concentrated feed, an improvement of preventive disease controls and biosecurity measures, and the use of technology to exhaustively control in-house environmental conditions [3]. In this context, ventilation of poultry houses plays a critical role to ensure appropriate indoor conditions for achieving a high animal productivity (growth and food conversion) and a low mortality [4–6].

The dominant ventilation system in modern broiler houses uses forced ventilation, mainly through negative-pressure systems. Ventilation design is based in three basic principles: the rate of air exchange, air distribution and air velocity range at the animal level [7–9]. However, the design of ventilation systems for poultry housing has seen a certain amount of development by trial and error in the absence of precise guidelines [10]. Therefore, it seems that further research is necessary to establish standardised protocols to design ventilation systems for poultry houses. The air exchange rate is calculated according to animal age and number in the building and is achieved with exhaust fans. The number of fans installed and operated will depend on ventilation needs and the performance of each fan. The air exchange rate must ensure a proper control of indoor temperature as well as an effective evacuation of air moisture and air pollutants [8]. On the contrary, the uniform distribution of air inside the house and the air velocity at animal level depend mainly on farm design and operation factors which interact in a complex way. Design factors (building geometry and location) and also operational factors (fan operation, adjustment of air inlet openings and pressure drop) become essential to define an optimal ventilation system [7–9]. However, the distribution of air may be affected under field conditions by unplanned openings (open doors and windows as well as cracks in walls or ceilings), bad adjustment of openings or impaired performance of exhaust fans caused by equipment ageing, bad maintenance or changes in electricity supply [7,11].

Some authors [12] have indicated that poultry farms with inadequate ventilation systems suffer from higher mortality rates when the indoor air is hot, humid and still in the zones occupied by animals. Even more, it has been reported that chicken's welfare is more influenced by the ventilation system than by the animal stocking density [13]. For this reason, the inappropriate design or malfunctioning of ventilation systems can enhance the occurrence of lethal environmental conditions within production buildings, thus leading to significant economic losses. Moreover, even well designed and operated buildings may be insufficient to cope with extreme circumstances. In this context, massive deaths of approximately 500,000 birds occurred in 2003 because of heat stress in the Valencian Community (Spain), a region with an approximate stock of 9,000,000 birds [14].

To assess the design and operation of ventilation systems in livestock houses direct measurements with appropriate instrumentation and measurement protocols are required. Alternatively, there is an increasing use of computational fluid dynamics (CFD) to indirectly evaluate ventilation systems in agricultural systems [15–20]. However, this indirect method also needs verification and validation (V&V), and thus using adequate instrumentation is also necessary. This instrumentation must allow simultaneous measurement of air velocity at different locations inside the house, but at the same time must be precise enough in the usual range of air velocity in broiler houses, which is normally lower than 3 m/s. Current commercial instrumentation systems, however, are not thought to evaluate ventilation systems of commercial farms because they normally measure only point values and their measurement thresholds are higher than the usual air velocity found in the farms. Furthermore, complex measurement systems should be avoided. One author [21] indicated that an instrumentation operator may produce distortions in the airflow inside the farm and its use may be unpractical under field conditions.

Recent studies have focused on the use of electronic instrumentation and sensors in farms. Some authors have developed systems to measure ventilation rates in livestock buildings, which are based on different sensors. So, turbinemeters have been used to determine the ventilation rate in livestock buildings [22] or a portable anemometer to determine the fan performance curve [23]. One study [24] implemented an instrumentation system for performing environmental measurements in broiler and swine housing, whereas another study [25] used an ultrasonic anemometer to measure the air velocity in animal-occupied zones in a swine farms. Another interesting study used hot-wire anemometry to measure the air velocity based on monitoring thermal losses in a heated measuring element [26]. However, in all cases, research until now has recorded only measurements taken at one or a few points and not addressed long term measurements using a large number of sensors.

Recently, a basic system for measuring temperature and air velocity in poultry houses was described [27], which has been used in later experiments [17]. The same authors suggested that the described measurement system could be used as a basis to develop a measurement system equipped with a larger number of sensors fulfilling the essential premise of simultaneous measurement at multiple points. To achieve this premise, the time delay between two consecutive acquisitions needs to be minimized and a multiplexing system arises as an essential element in the design of this ideal measurement system. A multiplexer allows for data acquisition in a quasi-simultaneous regime at different locations including animal level and other heights, minimising potential distortions of airflow inside the farm.

It must be considered that the airflow inside a mechanically ventilated building is turbulent by nature. The presence of animals intensifies this internal turbulent atmosphere, creating sudden changes of environmental parameters both in time and space. An instrumentation to evaluate the indoor climate of a livestock building must therefore receive as many input signals per time unit as possible, from a large number of widely distributed measuring locations, particularly from zones occupied by animals [21,27,28]. As indicated above, it is also necessary to measure differential pressure because of its critical influence on ventilation performance of a mechanically ventilated farm.

The main objective of this study was to develop a multisensor system to evaluate the design of the ventilation system in broiler houses. The system was designed to measure simultaneously air velocity, temperature and differential pressure with different sensors. This system was calibrated and then tested under farm conditions and may serve as a useful tool to evaluate the indoor environment of poultry farms, as affected by farm design, for troubleshooting, and as a V&V system of CFD simulations.

## 2. Materials and Methods

In this section, the developed measurement system, as well as the methodology followed for its field validation, will be described.

### 2.1. Measurement System Development

#### 2.1.1. General Description

A configurable multi-sensor device aimed at measuring air velocity, temperature and differential pressure in multiple locations at the same time was designed and built. The system consisted in a portable PC (Pentium III, 64 Mb RAM) and a National Instruments Corporation (Austin, TX, USA) DAQCARD 6024E data acquisition card with 16 analogue inputs and a maximum sampling rate of 200 kS/s. Its absolute accuracy at full scale was 10.568 mV. As 16 channels were less than those needed, a central multiplexor data collection module was designed. The multiplexing modules were able to concentrate eight signals into a single channel. Therefore, the system extended the 16 channels of the acquisition card to a maximum of 128 signals. To reduce interferences, all information was sent in current mode instead of voltage mode. The card also had eight digital input-output channels, which were used to control multiplexing units.

**Figure 1.**
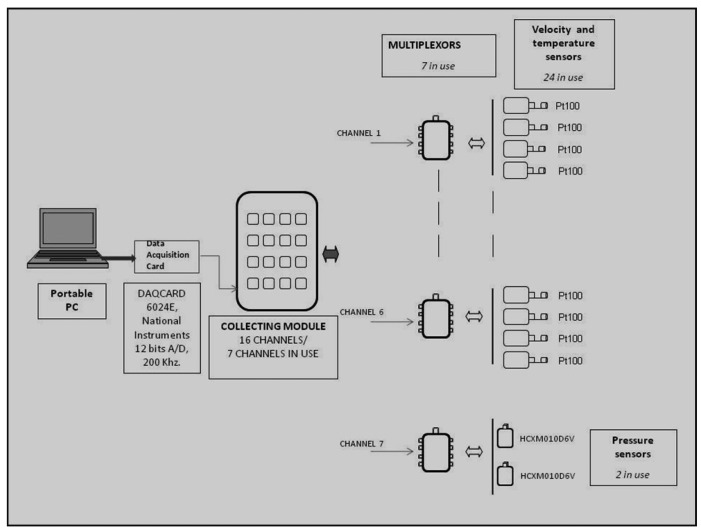
Scheme of the measurement system.

In this paper, we describe a system adapted to operate with 24 air velocity sensors, 24 temperature sensors, two differential pressure modules and seven multiplexers. A schematic of the system is shown in Figure 1.

Data was acquired in the PC by using specifically developed software. This software was based on the National Instruments Corporation LabVIEW 8.2 platform [29]. The software was able to acquire and monitor signals from sensors, as well as control the multiplexing and demultiplexing functions.

Two programs were developed in LabVIEW. One to monitor all the sensors used in the installation at real time, and another one that shows on the screen the time evolution of the sensors and can record data on the PC at the programmed rate. In this experiment, data of all sensors is taken every five seconds and kept for an average of 120 readings (every ten minutes).

#### 2.1.2. Temperature Material and Circuit

**Figure 2.**
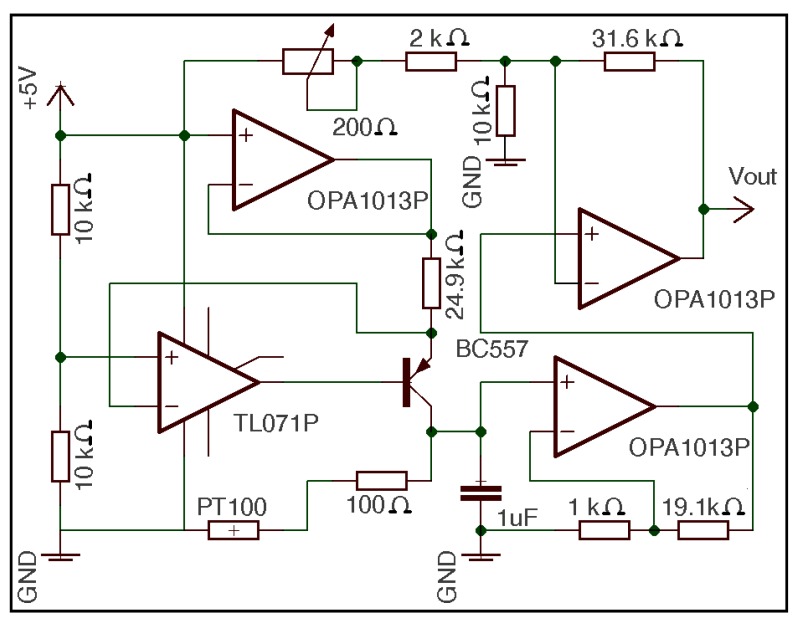
Circuit of the temperature sensor.

A platinum resistance temperature detector (RTD) thin film detector Pt100 (Omega, Inc., Stamford, CT, USA) printed on a ceramic substrate [30] was chosen as the temperature sensor; the technical characteristics of the device are consistent with Deutsches Institut für Normung (DIN)-43760 and British Standard (BS)1904. Figure 2 shows the circuit of the temperature sensor. It is used to linearise the response of the Pt100 and to regulate the zero offset by the variable resistor of 200 Ω.

#### 2.1.3. Temperature Calibration

In order to calibrate temperature sensors, a Fluke Corporation (Everett, WA, USA) temperature calibrator Fluke-724 that simulates a Pt100 was used. The sensor was disconnected from the electronic circuit and connected to the simulator. It was also connected to the acquisition system in the temperature module to measure the output voltage from the different temperatures simulated using the Fluke-724. Calibration temperature ranged from 0 °C to 44 °C. This range is found within the common range of temperatures in commercial poultry farms. For each of the 24 sensors, two consecutive calibrations were conducted to study potential hysteresis. The first calibration lay between 0 °C and 44 °C, whereas the second lay between 44 °C to 0 °C. Each calibration was performed at 2 °C intervals within each range. Therefore, a total of forty six temperature values were used to calibrate each sensor.

For each calibration, the output potential (*U_t_*) was measured as a linear function of temperature, including a quadratic term following Equation (1). The quadratic term was used to account for those cases where the linear effect varied with increases in temperature:

(1) $$U_{t}=\alpha +\beta \times T+\gamma \times T^{2}$$

To detect differences between sensors a unique regression analysis integrating all sensors with dummy variables was used following the model:

(2) $$E(U_{t})=\propto_{0}+\beta_{0}\times T+\gamma_{0}\times T^{2}+\sum_{i=1}^{i=n-1}\propto_{i}\times S_{i}+\sum_{i=1}^{i=n-1}\beta_{i}\times S_{i}\times T+\sum_{i=1}^{i=n-1}\gamma_{i}\times S_{i}\times T^{2}$$

where:

*E(U_t_)*: Mean value of the measured potential *U_t_* (volts) in both calibrations with the multimeter.
*T*: Air temperature (°C).
*S_i_*: Sensor *i* (dummy variable) that takes 0 and 1 values; for any specific sensor, the variable takes a value of 1 and 0 in all cases (1 for the sensor that corresponds to the observation and 0 for the rest of the sensors).
*α_0_*: Independent coefficient of regression.
*β_0_*: Regression coefficient of variable temperature (*T*) simulated at calibrator “Fluke 724” (in °C).
*β_i_*: Regression coefficient of the interaction between variables *T* and *S_i_*.
*γ_0_*: Regression coefficient of the variable for the square of the temperature (*T^2^*).
*α_i_*: Regression coefficient of the variable sensor (*S_i_*).
*γ_i_*: Regression coefficient of the interaction between variables *T^2^* and *S_i_*.

The dummy variables (*S_i_*) had to be created so that they assume a value equal to the number of variables minus 1; thus, a reference sensor was used to determine all variables. If the sensor assumes a value of 0, the rest assume a value 1 with respect to one of these variables [31]. Differences between sensors in the model were detected in three ways: changes in the intercept (*α* coefficients), changes in the slope (*β* coefficients) and the square coefficients (*γ*).

The model in Equation (2) provided the equation of the reference sensor (Equation (3)) and the coefficients of the other sensors (Equation (4)):

(3) $$E(U_{t})=\propto_{0}+\beta_{0}\times T+\gamma_{0}\times T^{2}$$

(4) $$E(U_{t})=\left(\propto_{0}+\propto_{i}\right)+\left(\beta_{0}+\beta_{i}\right)\times T+(\gamma_{0}+\gamma_{i})\times \;T^{2}$$

This analysis was performed with the PROC REG procedure of SAS [32]. Maximum and minimum differences between measures and estimated observations were taken as a practical criterion of the accuracy of the model.

#### 2.1.4. Air Velocity Material and Circuit

Among the different available technologies used to measure air velocity [33], hot-wire anemometry was chosen because of its various advantages. The basic principle of hot-wire anemometry is very simple: a fluid (in this case air) crosses an (electrically) heated wire at a constant temperature; thus, an energy balance can be established between the power supply to the heated wire and the dissipation, which is proportional to the air velocity.

**Figure 3.**
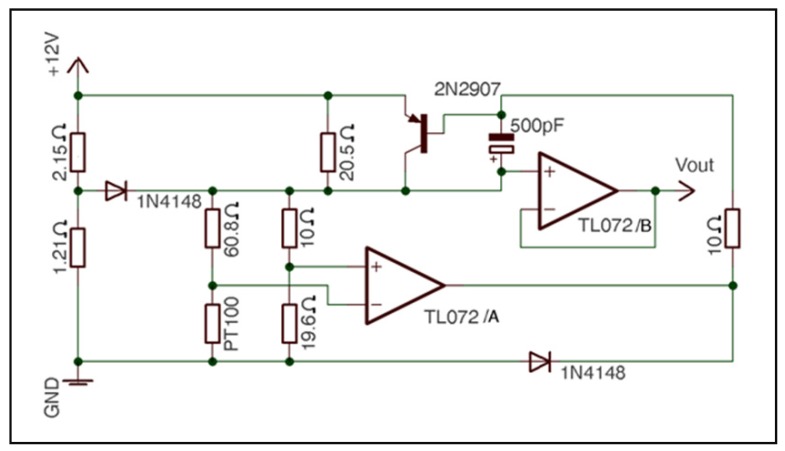
Circuit of the air velocity sensor.

Based on hot-wire anemometry, an RTD was chosen as air velocity sensor. An RTD consists of a thin wire, sheet or metallic component that is generally supported by a ceramic. In this case, the RTD featured a thin platinum piece, whose processing offered a resistance of 100 Ω at 0 °C; thus, the RTD was referred to as Pt100. In fact, Pt100 has great advantages: minimal thermal mass, the ability to detect small mass velocity, mechanical robustness and no moving components, easy mounting, very fast response time, ability to perform a simple electronic analysis, the best price-performance ratio, good repeatability and great stability over time [34]. For these reasons, the same sensor as in temperature determinations (platinum resistor Pt100, printed on a ceramic substrate, thin film detector (TFD), Omega Inc. [30]) was chosen for the velocity measurements. The electronic circuit of the air velocity sensor is shown in Figure 3.

The circuit operates as follows: the resistors of 10 Ω, 60.18 Ω and 19.6 Ω and the Pt100 form a Wheatstone bridge. To make this bridge balanced, the resistance of the Pt100 should be 119.168 Ω which is equivalent to a temperature of 49.41 °C. If the bridge becomes unbalanced, the operational amplifier TL072/A and the transistor 2N2907 act until the bridge is balanced again.

#### 2.1.5. Air Velocity Calibration

The equation governing the thermal equilibrium between the heating of the sensor to a constant temperature and the dissipation of the air is:

(5) $$\frac{dE}{dt}=W-H$$

where *E* is the thermal energy stored in the sensor, *W* is the electrical power applied to it and *H* is the energy dissipated to the surroundings.

Under quasi-stationary conditions, the thermal energy stored is constant, so:

(6) 0=W-H

(7) $$W=\overset{U^{2}}{\;}/\underset{R(T_{W})}{\;}$$

where *R(T_w_)* is the resistance of the Pt100 at a constant temperature (*T_W_* = 49.41 °C), and *U* is the voltage applied to the Pt100.

Assuming convection is the main mode of heat transmission (neglecting radiation and conducting losses):

(8) $$H=h\cdot A\cdot (T_{w}-T_{f})$$

where *h* is the film coefficient of heat transfer, *A* is the sensor surface and *T_f_* is the temperature of fluid. In a forced convection regime, coefficient (*h*·*A*) may be expressed as:

(9) $$h\cdot A=a+b\cdot V_{f}^{n}$$

where *a* and *b* are constants dependent on the fluid, in this case air, and *V_f_* is the velocity of the fluid. Replacing and rearranging Equation (6) produces an expression that relates voltage *U*, the fluid velocity *V_f_*, the wire temperature *T_W_* and the fluid temperature *T_f_*, resulting in:

(10) $$\overset{U^{2}}{\;}/\underset{R(T_{W})}{\;}=\left(T_{w}-T_{f}\right)\cdot \left(a+b\cdot V_{f}^{n}\right)$$

In this paper, *n* is assumed to be 0.5 [35–38]. Grouping and transforming the constants (*a, b, R(T_W_)*), results in Equation (11), known as King's Law:

(11) $$\frac{U^{2}}{T_{w}-T_{f}}=\delta +\lambda \;V_{f}^{0.5}$$

**Figure 4.**
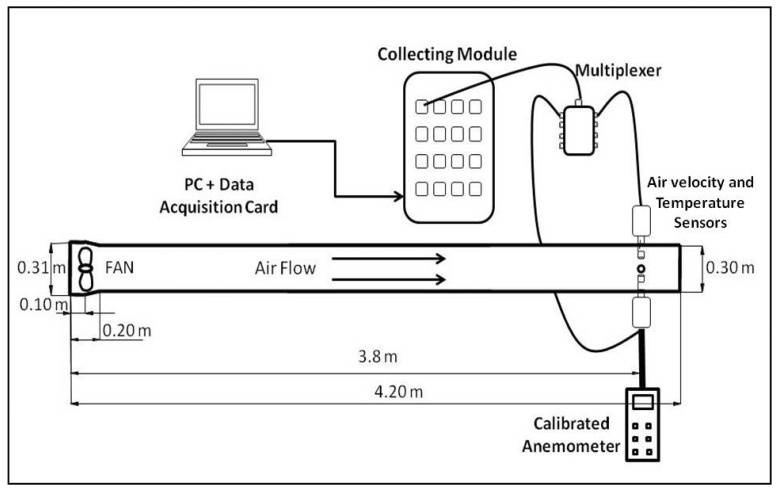
A scheme of the wind tunnel showing the position of the air velocity and temperature sensors.

A wind tunnel was designed to calibrate the velocity sensors (Figure 4).

Fifteen velocity values were measured in the wind tunnel within the range from 0.1 to 4.5 m/s. These velocities were obtained varying the fan power. A calibrated Testo 425 hot-wire anemometer was used to obtain reference values (Testo Inc., Sparta, NJ, USA; error 0.03 m/s + 5% of the measured value) [39].

The calibration procedure was performed as follows [40] according to the designed circuit (Figures 2 and 3) and from Equation (11). So, if we called:

(12) $$y=\frac{U_{v}^{2}}{T_{w}-T_{f}}$$

(13) $$x=\sqrt{V_{f}}$$

The follow Equation (14) results:

(14) y=δ+λ⋅x

A least-square algorithm is used to obtain the coefficients of the linear regression (*δ, λ*) between the reference air velocity (*V_f_)* measured with the reference anemometer and the terms included in *y* (Equation (12)), so that *T_W_*, the temperature of the wire that is fixed by the electronic circuit (*T_W_* = 49.41 °C), the output voltage of the velocity (*U_v_*) and temperature (*U_t_*) sensors was measured; in turn, the output voltage of the temperature it used to calculated the sensor temperature (*T_f_*) through its corresponding calibration, as explained before. In conclusion, a collection of values (*U_v_, V_f_, T_a_*) was measured simultaneously while controlling the fan power.

MATLAB software [41] was used to create a program to sum up the whole process, and then calculate the regression coefficients *δ* and *λ* of the Equation (14) by the PROC REG procedure of the SAS program [32]. A curve of specific calibration was obtained for every module sensor.

Once all regressions were obtained for the different sensors, in order to ascertain the validity of the calibrations a linear regression analysis between the velocity measured by the anemometer (*V_f(real)_*) and the estimated velocity (*V_f(e)_*) by the calibration procedure described, done as [40]. The statistical model used in order to compare calibration curves was a linear regression using the PROC REG procedure of SAS [32] too:

(15) $$E(V_{f(\text{real})})=\propto_{0}+\beta_{0}\times V_{f(e)}+\sum_{i=1}^{i=n-1}\propto_{i}\times S_{i}+\sum_{i=1}^{i=n-1}\beta_{i}\times S_{i}\times V_{f(e)}$$

where:

*E(V_f(real)_)*: Mean air velocity measured with the hot-wire anemometer (m/s).
*V_f(e)_*: Estimated velocity determined by the calibration procedure for the sensors.
*S_i_*: Sensor i (dummy variable) that take 0 and 1 values; for a specific sensor, the variable takes a value of 1 and 0 in all cases (1 for the sensor that corresponds to the observation and 0 for the rest of the sensors).
*α_0_*: Independent coefficient of regression.
*β_0_*: Regression coefficient of the *V_f(e)_* variable.
*α_i_*: Regression coefficient of the variable *S_i_*.
*β_i_*: The regression coefficient of the interaction *S_i_* by *V_f(e)_*.

The model provided differences for the reference sensor (Equation (16)) and the others sensors (Equation (17)):

(16) $$E(V_{f(\text{real})})=\propto_{0}+\beta_{0}\times V_{f(e)}$$

(17) $$E(V_{f(\text{real})})=\left(\propto_{0}+\propto_{i}\right)+\left(\beta_{0}+\beta_{i}\right)\times V_{f(e)}$$

Maximum and minimum differences between measures and estimated observations were taken as a practical criterion of the accuracy of the model.

#### 2.1.6. Differential Pressure Module

**Figure 5.**
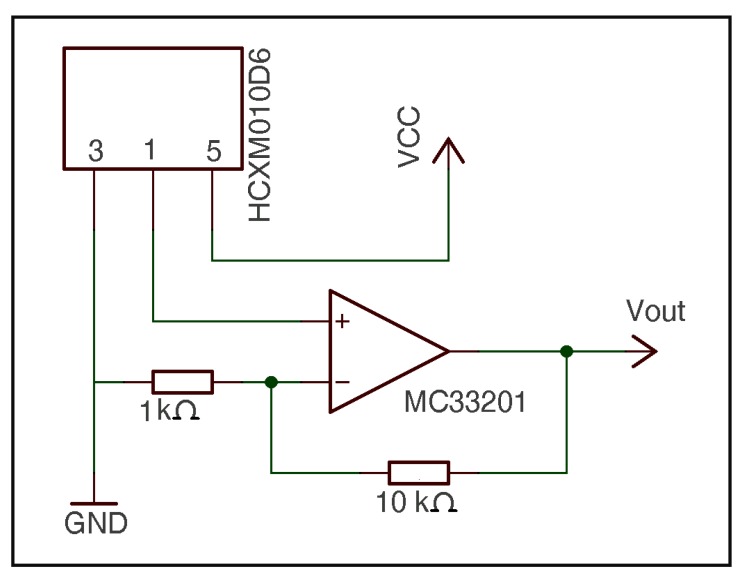
Differential pressure electronic circuit.

A sensor with a range between 0 and 100 Pa was selected as the operating conditions rarely exceed 60 Pa. A HCXM010D6V differential pressure-sensing module based on a pre-amplified silicon gauge pressure sensor (Sensortechnics Inc., Puchheim, Germany); nonlinear and hysteretic error <0.5% [42]) was used. This module has previously been used in mechanically ventilated poultry farms [17]. An electronic circuit was designed to obtain a range between 0 and 100 Pa by a non-inverting, operational amplifier with a gain of 11, as shown in Figure 5.

The differential pressure modules were calibrated using a differential manometer (U-shaped tube filled with distilled water), a dropper and the data acquisition system. The calibration was performed with water at 4 °C because, at this temperature, the water density is 1 g/mL. Different output voltages related to the displacements of the liquid column were measured. These displacements were observed along a vertical ruler using a magnifying glass. Moreover, in order to improve the precision, the volume of water injected was registered and counted drop by drop. The maximum range of calibration (100 Pa) corresponded to one centimetre of displacement and 13 drops of 100 μL. The calibration equation was as follows:

(18) $$\text{U}=\propto_{0}+\beta_{1}\times dP$$

where:

*U*: Measured voltage (in volts).
*α_0_*: Independent coefficient of regression.
*β_1_*: Regression coefficient of independent variables.
*P*: Pressure (in Pa) calculated from the displacement of the water column.

Through a regression analysis using SAS [32], the regression coefficients were estimated. A regression equation was developed for each of the two sensors. As with air temperature and air velocity sensors, maximum and minimum differences between measures and estimated observations were taken as a practical criterion of the accuracy of the model.

### 2.2. Field Experiments

#### 2.2.1. Assay Building

The system was tested in a commercial broiler farm which was selected for its location (Villarreal, Castellón, Spain) and climatic conditions that are representative of the Mediterranean region. These climatic conditions are characterized by high temperatures and high relative humidity (e.g., >30 °C, >70% RH).

The farm featured a mechanical cross-ventilation system. The dimensions of the building were: length, 110 metres; width, 12.60 metres; sidewall height, 2.6 metres; slope cover, 21.53%. Sixteen exhaust fans were installed: nine large fans with a diameter of 1.28 m (Gigola & Riccardi, Cazzago San Martino, Italy, model Gigola ES-140, with a power consumption of 0.74 kW and a nominal ventilation flow of 34,956 m^3^·h^−1^ to ΔP = 0 Pa) and seven 0.68 m diameter small ones (Ziehl-Abegg A.G., Küzelsau, Germany, model FC063-6D, power consumption 0.58 kW and nominal ventilation flow 12,750 m^3^·h^−1^ to ΔP = 0 Pa). The farm was equipped with 66 Tuffigo^©^ air inlets (Quimper, France, model Kan'Air, 0.795 × 0.24 metres placed at 1.51 metres height) controlled by an automatic system for automatically management in three groups of 22 inlets. The building was empty during the experiments to avoid possible interference due to the presence of animals.

#### 2.2.2. Measurement Conditions (Scenarios)

To test the measurement system, four different boundary conditions were established: (I) 30 Pa using only large fans, (II) 38 Pa working all fans, (III) 50 Pa working large fans and (IV) 50 Pa working all fans). Two sections were studied, one (Section A) was located near one extreme of the building, whereas the second (Section B) was in the centre. Table 1 shows trial scenarios.

**Figure 6.**
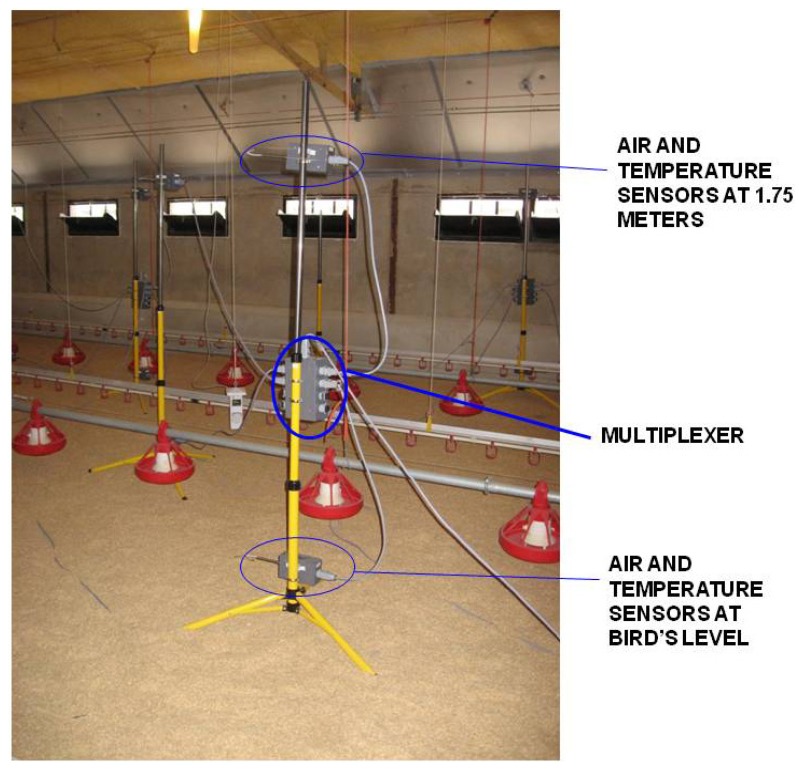
Tripod with a multiplexer at its centre and two air velocity and temperature sensors at the level of the birds (0.25 metres) and at 1.75 metres.

For each scenario, all 24 air velocity and temperature sensors, two differential pressure sensors and seven multiplexers were placed on 12 tripods; two sensors were placed on each tripod: one of them at the level of the birds (0.25 metres) and the other at a height of 1.75 metres. A detail of a tripod when measuring in the building is shown in the photograph, Figure 6.

**Figure 7.**
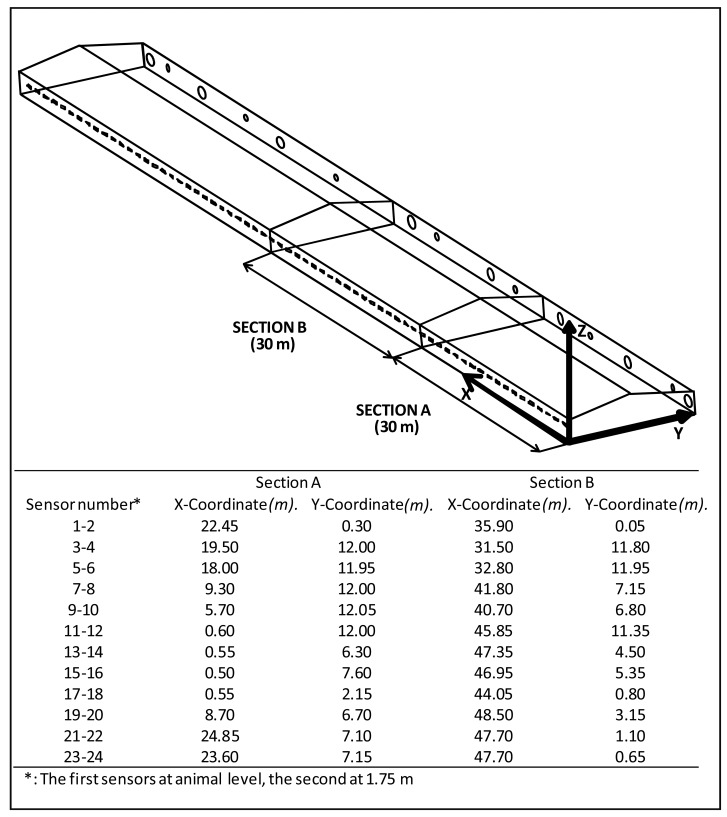
Location of the measurements in the two sections of the poultry farm.

To sum up, eight trials were conducted (two sections with four boundaries) with 12 measurement positions as indicated in Figure 7. The location of the sensors was chosen according the situation of inlets and fans and considering where the farmer had observed any anomaly such as a greater or lesser concentrations of chicken or increased mortality. Acquisition time for each trial was 10 minutes. As the system was programmed for measuring each 5 seconds, each value was the mean of 120 data.

For all tests, the two differential pressure sensors were used to control the opening of the inlets and performance of the fans precisely.

#### 2.2.3. Statistical Procedures

In order to study the effect of factors involved in the performance of the system under field conditions, an analysis of variance following the next model was developed:

(19) $$Y_{ijkl}=\mu +Z_{i}+B_{j}+H_{k}+S_{l(k)}+{\left(Z\times B\right)}_{ij}+{\left(Z\times H\right)}_{ik}+{\left(B\times H\right)}_{jk}+{\left(Z\times B\times H\right)}_{ijk}+{\left(Z\times S\right)}_{il(k)}+{\left(B\times S\right)}_{jl(k)}+{\left(Z\times B\times S\right)}_{ijl(k)}$$

where:
*Y_ijkl_*:*Air velocity measured in the Section i at conditions j at k height by sensor l*.*μ:**Overall mean*.*Z_i_*:*Measurement Section (2)*.*B_j_*:*Boundary conditions (4)*.*H_k_*:*Height of sensor (2)*.*S_l(k)_*:*Sensor (24) hierarchical to height*.*(Z × B)_ij_*:*Interaction Section-Boundary (8)*.*(Z × H)_ik_*:*Interaction Section-Height (8)*.*(B × H)_jk_*:*Interaction Boundary-Height (8)*.*(Z × B × H)_ijk_*:*Triple interaction Section-Boundary-Height (16)*.*(Z × S)_il(k)_*:*Interaction Section-Sensor (48)*.*(B × S)_jl(k)_*:*Interaction Boundary-Sensor (96)*.*(Z × B × S)_ijl(k)_*:*Triple interaction Section-Boundary-Sensor (residual term of the model)*.

Numbers in parentheses indicate number of factors. To study these effects, all factors were considered to be at random. The model was analysed by the GLM procedure of SAS systems [32].

## 3. Results and Discussion

### 3.1. Sensor Calibration

#### 3.1.1. Temperature Calibration

Table 2 presents the regression coefficients resulting from applying the regression model in Equation (2). Five regression equations were obtained for different groups of sensors according to the nature of the calibration curves (Equations (20) to (24)). In all cases the regressions showed high significance (P < 0.001) and goodness fit (R^2^ = 0.99). The first line in Table 2 (Equation (20)), represents the reference sensor's regression coefficients after applying Equation (3), *i.e., α_0_* = 2.00, *β_0_* = 0.152 and *γ_0_* = 0, and thirteen other sensors that were not significantly different from it, *i.e., α_i_* = 0, *β_i_* = 0 and *γ_i_* = 0, after applying Equation (4).

The difference between Equations (20) and (21) was the temperature coefficient, representing the slope of the regression line. The four sensors grouped in Equation (21) showed a slightly lower slope than sensors grouped in Equation (20). Equations (23) and (24) only grouped one sensor each and showed a higher temperature coefficient (slope) (Equation (23)) or lower intercept (Equation (24)) than sensors grouped in Equation (20). Equation (22) grouped four sensors and showed the most different coefficients. Equation (23) showed a lower intercept and a higher slope than sensors grouped in Equation (20), but a negative quadratic term. The effect of the quadratic term indicated that an increase in the temperature reduced the slope of the regression equation. In this case, clearing the temperature produced a second-degree polynomial equation.

For Equations (20) and (24) the maximum error measured was 0.33 °C and the minimum was <0.01 °C, for Equation (21) the maximum error measured was 0.07 °C and the minimum was <0.01 °C, for Equation (22) the maximum error measured was 0.05 °C and the minimum was <0.01 °C and finally for Equation (23) the maximum error measured was 0.32 °C and the minimum was <0.01 °C. As in all cases the minimum error was lower than the reading of the simulator (0.01 °C), we used these minimum errors for estimations. In all cases a very low error hysteresis value was obtained (±0.0227%).

#### 3.1.2. Air Velocity Calibration

**Figure 8.**
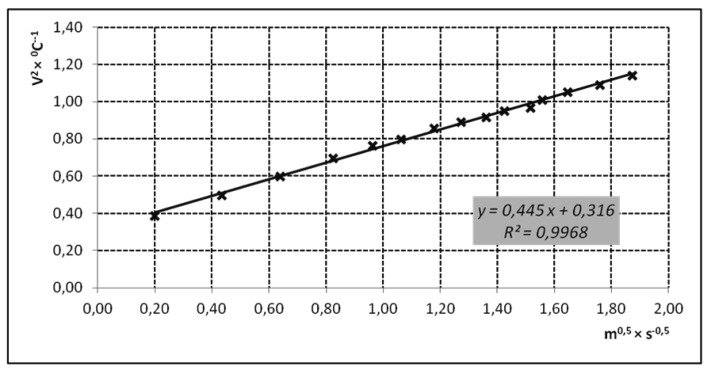
Regression curve of a velocity sensor calibration.

Figure 8 shows an example of the result of the calibration of an air velocity sensor, where the regression parameters of Equation (11) are shown.

The estimated velocities (*V_f(e)_)* (m/s) were obtained from the regression of each sensor. Comparing these estimated velocities with real velocities *V_f(real)_* (m/s), using the model of Equation (15), we obtained a high significance (P < 0.001) with a high goodness of fit (R^2^= 0.99), but no differences between sensors were detected. Consequently, only one calibration curve was obtained for all velocity sensors:

(25) $$E(V_{f(\text{real})})=-0.01431+0.9956\;V_{f(e)}$$

The maximum error measured was 0.018 m/s, and the minimum was 0.002 m/s. Since these errors were smaller than the error of the anemometer (0.03 m/s + 5% of the reading), we used the anemometer error for calculations.

#### 3.1.3. Differential Pressure Calibration

The calibration results for the differential pressure sensors shows two similar calibration curves:

(26) $$\text{Sensor}1:U=5.6911+0.045dP(R^{2}=0.99;p<0.001)$$

(27) $$\text{Sensor}2:U=5.6381+0.0465dP(R^{2}=0.99;p<0.001)$$

The regressions were similar with respect to their intercepts and slopes. To obtain *dP* from the measured voltage (*U*), only Equations (26) and (27) had to be changed. The maximum error measured was 0.41 Pa, and the minimum was 0.22 Pa for differential pressure sensor 1 and the maximum error measured was 0.29 Pa, and the minimum was 0.20 Pa for differential pressure sensor 2.

### 3.2. Field Experiment

Considering all scenarios and sensors, 23,040 data values of air velocity were measured with the designed system. Table 3 shows the results of the ANOVA analysis. The variable sensor resulted not significant, as section, boundary and height and some interactions. Only the interactions “Section by Sensor (Height)” and “Boundary by Sensor (Height)” were significant.

Table 4 shows the obtained air velocity values according the different variables (Section, Boundary and Height) and some interactions. These values shows that the air velocities achieved in all boundaries are very homogeneous, the minimum value is 0.37 ± 0.30 m/s (Section B, Boundary *I*) and the maximum value is 0.80 ± 0.31 m/s (Section B, Boundary *III*) although there are peak measurements (not reflected in Table 4) between 0.06 m/s to 3.52 m/s.

### 3.3. Discussion

#### 3.3.1. Measurement System Development

The measurement system developed in this study was able to integrate calibrated sensors of temperature, air velocity and differential pressure and operated succesfully in different conditions in a mechanically-ventilated poultry farm.

It is currently accepted that wireless sensor networks can be applied to monitor environmental parameters in agricultural systems [44,45]. However, wired sensors were used because they were considered more appropriate for our measurement needs than wireless sensors. In this sense, hot-wire air velocity sensors operated with a frequent data collection interval (10 minutes in this work) which imply a high energy consumption. Therefore the batteries required for a wireless system are not able to guarantee energy supply for long-term measurements. This wired acquisition system can operate during one whole rearing cycle of broilers (6 to 7 weeks) with minimum maintenance, which avoids disturbing the normal operation in the farm. Similar wired systems to measure environmental parameters have also been used recently [46] obtaining succesful results.

In addition, this measurement system can receive the environmental signals without the physical presence of a technician (avoiding then the interference on measured values). This is due to its large data storing and adquisition capacity as well as its autonomy in terms of energy consumption [21].

Regarding to sensors nature, air velocity and temperature are hot-wire type sensors and RTDs, respectively. They were chosen based on their advantages, such as their robustness observed for similar uses [27]. On the other hand, there is still a need to test differential pressure sensors in mechanically ventilated poultry farms [17].

According to the results of this work, a single calibration curve was obtained for all velocity sensors. The error of the calibration curve was lower than the measurement error of the anemometer used for the calibration (±5% of reading) which indicates a good agreement among all sensors. On the contrary, several calibration curves were required for different temperature sensors. The results indicated that the sensors in this study could be classified into five statistically different groups, but differences between the five calibration equations were irrelevant in practical terms. This indicates that slight differences among sensors could arise from differences in the fabrication process or components. Regarding the calibration of differential pressure sensors, similar calibration curves were obtained, as expected considering the nature of these sensors.

The use of dummy variables for sensor calibration has been an innovative method for this purpose. So, using this tool allows obtaining optimum number of calibration curves according to statistical criteria. Ideally, a single calibration curve should be used, nevertheless a variety of factors including differences in fabrication, components or welds, make it not always possible in practice. Moreover, the precision required for measurement systems plays an crucial role, and using this methodology sensors can be grouped in homogeneous groups when small differences are observed. As indicated above, this is the case of temperature sensors in this study.

#### 3.3.2. Field Experiments

The sensors and the measurement system were tested in a commercial farm located in Eastern Spain. It is important to remark that despite commercial broiler farms do not follow any standard in terms of geometry or construction design, according to a wide knowledge base regarding the general construction characteristics of poultry farms in the region [47], this farm had typical dimensions and can therefore be considered representative of typical mediterranean broiler buildings.

Although the tests conducted in this study were performed in an empty broiler house, the configuration of the system and materials used, make the system robust enough to resist the aggressive environmental conditions (e.g., dust, high relative humidity, and gas concentration), occurring in buildings during animal rearing. Nevertheless, air velocity changes when psychometric conditions are modified by fluctuations of temperature or air density must be kept in mind. In this sense, in occupied farms, indoor environmental boundary conditions are more complex than in an empty farm. The reasons for this difference are the influence of broiler heat, chemical reactions of litter, as well as the refrigeration or heating systems. In any case, when using this system in occupied building, some improvements are recommendable. First, sensors placed at animal level must be protected (e.g., using a mesh) to avoid access by the animals. Second, it is also recommendable to include more sensors at a different level above the birds' heads in order to reduce the effect of the animals on measurements. Other practices such as frequent revisions and cleaning of sensors are also recommendable. Nevertheless, it would be necessary to test the system with animals to study the system's reliability.

In this paper, only air velocity data were presented and discussed since temperature and differential pressure conditions were similar for all situations investigated. In this regard, despite the fact that in this work only air velocity data were presented, in the case of occupied farms, when refrigeration and heating systems are operating or when significative differences between exterior and internal temperatures occur, additional studies on the results of temperature should be developed. Moreover, additional sensors (*i.e.*, humidity sensors, *etc.*) can be implemented at the other channels of the collecting module for wider studies, for example when measurement conditions take place under different environmental conditions.

According to the evaluation of effects on air velocity records, results obtained show that the variation between sensors was not significant, as expected according to the information obtained in the calibration procedure. Moreover, the interactions “Section by Sensor (Height)” and “Boundary by Sensor (Height)” were the only significant interactions. The lack of statistical significance of the variable “Sensor” in this simple effect indicates homogeneous behaviour of all sensors on average for the different sections and boundaries studied. However, the interaction “Boundary by Sensor (Height)” indicates that the effects of the air velocity changing the boundary conditions are not identical in all the sensors, *i.e.*, this interaction indicates that the differences between the boundaries that do not appear in the average because the factor is not significant (boundaries). In the same way, the interaction “Section by Sensor (Height)” indicates that the air velocity changes at each section is not identical in all sensors, *i.e.*, this interaction indicates the differences between sections that do not appear in the average because the factor is not significant (sections). These results are in accordance with the continuity equation for the case of infinite points [48], and they have important consequences in determining the locations of the sensors.

Regarding the performance of the ventilation system, when the best scenarios under which high mortality would be prevented during summer months was explored and a high value of the air velocity was not obtained at the level of the birds (a maximum of 0.80 ± 0.31 m/s in Section B, Boundary *III*). For this reason, it can be concluded that cross-mechanical ventilation is a good system for mild weather, but it is neccessary to explore other conditions of the ventilation system to prevent episodes of high mortality during summer months because this mechanical system of ventilation does not offer high air velocities at the level of the birds.

Moreover, it was interesting to note the great fluctuation in the values of air velocity in a mechanically ventilated poultry farm. As observed in the Table 4, the overall mean is 0.63 ± 0.54 m/s, the means by section are 0.59 ± 0.50 m/s (Section A) and 0.68 ± 0.58 m/s (Section B) and by 0.51 ± 0.70 m/s (Boundary *I*), 0.64 ± 0.37 m/s (Boundary *II*), 0.75 ± 0.39 m/s (Boundary *III*) and 0.64 ± 0.62 m/s (Boundary *IV*). This variability is due air turbulence in farm building [49] and the location of sensors [21,27]. Environmental parameters values obtained through this measurement system can be utilised for V&V procedures [50] of CFD works and futures studies.

## 4. Conclusions

An on-line computerized multisensor system for measuring air velocities, temperatures and differential pressure in multiple locations in poultry houses at the same time was designed and built. The system consisted in a laptop, a data acquisition card, a multiplexor module and a set of 24 air temperature, 24 air velocity and two differential pressure sensors. The system was able to acquire up to a maximum of 128 signals simultaneously at 5 second intervals.

The statistical procedures used to obtain calibration curves demonstrate the robustness of the system regarding temperature sensors. A single regression curve was obtained for 14 sensors, two curves for four sensors, and only two individual curves. Moreover, for air velocity sensors a single calibration curve was obtained. The regression error was smaller than the error of the reference anemometer.

Under field tests in a commercial broiler farm, the multipoint sensor system allowed for the measurement of a high number of input signals from different locations with minimum internal delay in acquiring signals. In terms of air velocity, the results allow to conclude that the variation among sensors was not significant. It also demonstrated to be robust and portable and could be used without presence of any operator which could disturb air velocity profiles.

The developed multisensor system can be used to obtain quasi-instantaneous fields of the air velocity and temperature, as well as differential pressure maps to assess the design and functioning of ventilation system and as a V&V system of CFD simulations.

## Figures and Tables

**Table 1.** Trial scenarios.

**Assay Section**	**Differential Pressure (Pa)**	**Ventilation Rate** ^1^ **m^3^/h**	**Operating Fans**	**Boundary Condition**
Section A	30	233,163	Large	*I*
	38	276,204	Large + Small	*II*
	50	193,518	Large	*III*
	50	250,472	Large + Small	*IV*
Section B	30	233,163	Large	*I*
	38	276,204	Large + Small	*II*
	50	193,518	Large	*III*
	50	250,472	Large + Small	*IV*

1 Ventilation rates were measured in each scenario as indicated by [43].

**Table 2.** Results of regressions of temperature calibrations.

**Numbers of Sensors**	**N (Data Number)**	**Regression Estimated Coefficient**			

		**Intercept**	**Temperature Coefficient**	**Square Temperature Coefficient**	**Equation**

		***α*_0_ + *α_i_***	***β*_0_ + *β_i_***	***γ*_0_ + *γ_i_***	
14	644	2.00	0.152	0	(20)
4	184	2.00	0.148	0	(21)
4	184	1.98	0.154	−0.000053	(22)
1	46	2.00	0.154	0	(23)
1	46	1.98	0.152	0	(24)

**Table 3.** ANOVA of the air velocity scenarios.

	**DF**	**Sum of Squares**	**Mean Square**	**F-Ratio**	**P-Value**
Section	1	0.37	0.37	3.06	0.7935
Boundary	3	1.41	0.47	12.19	0.9051
Height	1	>0.00	>0.00	0.00	0.9909
Sensor(Height)	22	16.82	0.76	1.05	0.4428
Section × Boundary	3	0.10	0.03	0.11	0.9471
Section × Height	1	0.35	0.35	0.46	0.5076
Boundary × Height	3	0.82	0.27	0.72	0.5760
Section*Boundary×Height	3	0.79	0.26	2.46	0.0700
Section × Sensor(Height)	22	13.46	0.61	5.71	<0.0001
Boundary × Sensor(Height)	66	14.69	0.22	2.08	0.0017
Residual	66	7.08	0.11	-	-
Total (corrected)	191	55.88			

**Table 4.** Air velocities in m/s (mean ± standard deviation) in the field experiment. The number of data is indicated in parenthesis.

**Boundary Conditions**	**Height**	**Section A**	**Section B**	**All**
I	0.25 m	0.62 ± 0.86 (12)	0.37 ± 0.30 (12)	0.50 ± 0.65 (24)
	1.75 m	0.37 ± 0.39 (12)	0.66 ± 1.00 (12)	0.52 ± 0.76 (24)
	All	0.50 ± 0.67 (24)	0.53 ± 0.74 (24)	0.51 ± 0.70 (48)
II	0.25 m	0.70 ± 0.35 (12)	0.71 ± 0.29 (12)	0.71 ± 0.33 (24)
	1.75 m	0.47 ± 0.32 (12)	0.68 ± 0.47 (12)	0.58 ± 0.41 (24)
	All	0.59 ± 0.35 (24)	0.70 ± 0.38 (24)	0.64 ± 0.37 (48)
III	0.25 m	0.78 ± 0.41 (12)	0.80 ± 0.31 (12)	0.79 ± 0.35 (24)
	1.75 m	0.63 ± 0.34 (12)	0.77 ± 0.50 (12)	0.70 ± 0.42 (24)
	All	0.71 ± 0.37 (24)	0.79 ± 0.41 (24)	0.75 ± 0.39 (48)
IV	0.25 m	0.42 ± 0.26 (12)	0.65 ± 0.59 (12)	0.54 ± 0.46 (24)
	1.75 m	0.72 ± 0.69 (12)	0.77 ± 0.87 (12)	0.74 ± 0.75 (24)
	All	0.57 ± 0.53 (24)	0.71 ± 0.71 (24)	0.64 ± 0.62 (48)
All	0.25 m	0.63 ± 0.53 (48)	0.63 ± 0.41 (48)	0.63 ± 0.47 (96)
	1.75 m	0.55 ± 0.47 (48)	0.72 ± 0.71 (48)	0.63 ± 0.61 (96)
	All	0.59 ± 0.50 (96)	0.68 ± 0.58 (96)	0.63 ± 0.54 (192)
